# *RHD* Genotypes in a Chinese Cohort of Pregnant Women

**DOI:** 10.3389/fgene.2021.752485

**Published:** 2021-12-14

**Authors:** Jianjun Zhang, Yan Zeng, Yuefeng Wang, Jiaming Fan, Haijiang Chen, Dan Yang, Xiaoliang Shi, Hualin Xu, Zimu Fu, Fang Sheng, Jie Xuan, Xiaoxi Pan, Zhiming Zhang, Liping Ai, Yue Zhang, Jingjing Pan, Jing Zhao, Mingming Wang

**Affiliations:** ^1^ Department of Blood Transfusion, Shaoxing Maternal and Child Health Hospital, Shaoxing, China; ^2^ Genetics Department, Shaoxing Maternal and Child Health Hospital, Shaoxing, China; ^3^ Department of Obstetrics and Gynecology, Shaoxing Maternal and Child Health Hospital, Shaoxing, China; ^4^ Department of Gynecological Protection, Shaoxing Maternal and Child Health Hospital, Shaoxing, China; ^5^ Tianjin Super Biotechnology Developing Co., Ltd., Tianjin, China; ^6^ Zhejiang Biosan Biotechnology Co., Ltd., Hangzhou, China; ^7^ BGI Genomics, BGI-Shenzhen, Shenzhen, China

**Keywords:** *RHD*, genotyping, intron, splice mutation, sequencing

## Abstract

*RHD* variants in D¯ Chinese pregnant women arose difficulties in management during pregnancy. Therefore, this study aims to precisely manage D¯ pregnant women by evaluating the spectrum of *RHD* mutations in D¯ pregnant women and getting insight into the possible rare alleles of *RHD.* A total of 76 D¯ pregnant women were analyzed by performing polymerase chain reactions with sequence-specific primers (PCR-SSP), the 10 *RHD* exons Sanger sequencing, *RHD* zygosity detection, and mRNA sequencing (mRNA-seq). About 40% of alleles are variations of *RHD,* including *RHD* 1227A homozygous, RHD-CE(2-9)-D, et al. Therefore, we developed a molecular diagnostic strategy for Chinese women, and most D¯ pregnant women can be diagnosed with this simple decision tree. After *RHD* genotyping for D¯ pregnancy women, we eliminated at least 15% unnecessary ante- and postpartum injections of Rh immunoglobulin (RhIG). As the first pedigree study and the first functional analysis under physiological conditions, mRNA-seq revealed that c.336-1G>A mutation mainly led to the inclusion of the intron 2, which indirectly explained the D¯ phenotype in this family. We also developed a robust protocol for determining fetal RhD status from maternal plasma. All 31 fetuses were predicted as RhD positive and confirmed the RhD status after birth.

## Introduction

The D antigen is one of the most immunogenic, diverse, and clinically crucial protein-based blood groups. Anti-D is still the leading cause of the hemolytic disease of the fetus and the newborn (HDN) (Flegel et al., 2009). Therefore, Rh immunoglobulin (RhIG) treatment was recommended for D¯ pregnant women for the prevention of HDN (Qureshi et al., 2014). However, *RHD* genotyping identified that approximately 40% of the D¯ pregnant women with weak, discrepant, or inconclusive D¯ phenotype are not candidates for RhIG treatment (Londero et al., 2020). Thus, *RHD* genotyping is critical for the precise management of D¯ pregnant women (Sandler et al., 2015). *RHD* genotyping is also crucial for avoiding alloimmunization in blood transfusion. Evidence has confirmed that some D¯ blood donors have variant *RHD* alleles, which might cause alloimmunization in D¯ recipients. Therefore, genotyping for *RHD* in D¯ donors was suggested as a routine procedure in blood centers (Krog et al., 2011; Perez-Alvarez et al., 2019).

*RHD* genotyping is more critical for D¯ Chinese people, for about 40% of them have *RHD* variants instead of *RHD* deletion (Colin et al., 1991; Zhang et al., 2019). However, few studies get insights into the mRNA product of *RHD* variations in China (Liu et al., 2010). Moreover, Most *RHD* variants studies rely on the bioinformatics tools in silico and plasmid construct (Fichou et al., 2015; El Wafi et al., 2017). Very little functional data under physiological conditions is available to characterize the effect of the mutation at the molecular level.

At the same time, due to the high frequency of *RHD* variants in D¯ Chinese pregnant women, the high-throughput method, automated real-time quantitative polymerase chain reaction (PCR), is not eligible for non-invasive prenatal testing (NIPT) for predicting fetal RhD status in D¯ Chinese people (Clausen et al., 2019). Therefore, a more flexible method needs to be developed for D¯ Chinese pregnant women.

Thus, to precisely manage D¯ pregnant women, this study elucidated the molecular basis of D¯ pregnant women in Shaoxing and established a simple molecular diagnostic strategy for Chinese D¯ pregnant women. In addition, we investigate the first family study and the first mRNA sequencing (mRNA-seq) for c.336-1G>A alleles under physiological conditions to analyze the mechanism of intronic mutation. We also developed a robust protocol for determining fetal RhD status from maternal plasma.

## Materials and Methods

### Test Subjects

This study was approved by the Shaoxing Maternal and Child Health Hospital (approval n. 277). A total of 76 RhD-negative pregnant women underwent polymerase chain reactions with sequence-specific primers (PCR-SSP), 10 *RHD* exons Sanger sequencing, *RHD* zygosity detection, and mRNA-seq between November 2018 and June 2020.

### Test Method

#### DNA extraction

Nucleic acid extraction or purification was performed using commercial reagents (Tianjin Super Biotechnology Developing Co., Ltd., Tianjin, China; Lot No.: 201808001). Sample DNA was extracted according to the instructions of the DNA Extraction Kit, and the DNA concentration was measured and diluted to a final concentration of approximately 30 ng/μL (A260/280: 1.6–1.9). If the samples could not be tested immediately, they were stored at -20°C.

#### Polymerase Chain Reactions with Sequence-Specific Primers

The Human Erythrocyte *RHD* Genotyping Kit (PCR-SSP) (Tianjin Super Biotechnology Developing Co., Ltd., Tianjin, China; Lot No. 190830001) was used to detect eight common *RHD* genotypes: *RHD*-positive*, RHD* deletion, RHD-CE(2-9)-D, DVa (Hus), DVI III, weak D15, DEL *RHD* 1227A homozygous, and DEL *RHD* 1227A heterozygous. Reaction parameters were set according to the Human Erythrocyte *RHD* Genotyping Kit instructions, and amplification was performed on a Hema 9600 Gradient Thermal Cycler (Hangzhou Bioer Technology Co., Ltd., Zhejiang, China). The method is briefly described as follows. The PCR amplification system consisted of dNTP-Buffer working solution 80 μL, Taq enzyme 0.8 μL, DNA 10 μL, and 10 μL of the above-mixed solution was added into each of the eight wells coated with primers. PCR amplification procedure: pre-denaturation at 96°C for 2 min; 96°C for 20 s, 68°C for 1 min, 5 cycles; 96°C 20 s, 65°C 50 s, 72°C 45 s, 10 cycles; 96°C 20 s, 62°C 50 s, 72°C 45 s, 18 cycles; Finally, it was extended at 72°C for 5 min. PCR products were visualized on a gel imager after 2.5% agarose gel electrophoresis, and the results were interpreted according to the instructions provided with the kit.

#### Zygosity Detection and Sanger Sequencing

*RHD* zygosity detection and the 10 *RHD* exons Sanger sequencing were commissioned to Tianjin Super Biotechnology Developing Co. (China). *RHD* zygosity was assessed by the PCR method. Two pairs of primers were designed to amplify the hybrid Rhesus box of *RHD-* (2,700 bp) and internal control (1009 bp), respectively. If a hybrid Rhesus box is detected, there is a complete deletion of the *RHD* gene. The Sanger dideoxy method performed direct Sequencing of all the 10 RHD exons and flanking intron regions. Sequence analysis was performed by DNAMAN v9 (Lynnon Biosoft Co., United States) and ChromasPro v1.2 (Technelysium Pty. Ltd., Australia), and the reference allele was RHD*01 (NG_007494.1)

#### *RHD* mRNA-seq

*RHD* mRNA-seq was commissioned to Beijing Beikang Medical Laboratory Co. (China). First, the total RNA of whole blood was extracted, and the mRNA with polyA tail was enriched by Oligo (dT) magnetic column. Subsequently, the obtained mRNA was randomly interrupted with divalent cations in NEB Fragmentation Buffer, and the library was constructed by the standard NEB library construction method. NEBNext® UltraTM RNA Library Prep Kit for Illumina® was used for the library construction. The first strand of cDNA was synthesized in the M-MuLV reverse transcriptase system with the fragmented mRNA as templates and random oligonucleotides as primers, and then the RNA strands were degraded with RNaseH.

Moreover, dNTPs were used to synthesize the second strand of cDNA under the DNA polymerase Ⅰ system. After purifying the double-stranded cDNA, end-repair, A-tailing, and ligation of sequencing adapter were performed. Next, the 250–300 bp cDNA was screened with AMPure XP beads, PCR amplification was performed, and the PCR product was purified again with AMPure XP beads to obtain the library. Qubit® 2.0 Fluorometer (Thermo Fisher Scientific, Walsham, United States) was used for preliminary quantification after the library was constructed. Then the library was diluted to 1.5 ng/μL, and Agilent 2,100 bioanalyzer was used to detect the insert size of the library. Then qRT-PCR was used to measure the effective concentration of the library whose insert size was in line with the expectation. Accurate quantification by qRT-PCR (the effective concentration of the library was higher than 2 nM) was to ensure the quality of the library. Then the library was pooled and finally analyzed on the Novaseq 6,000 sequencer.

#### Non-Invasive Fetal *RHD* Genotyping

Cell-free fetal DNA (cffDNA) extraction from maternal plasma (200 μL) was performed using the BGISP-300 (BGI, Shenzhen, China) and the Nucleic Acid Extraction (BGI, Shenzhen, China) kits. First, the DNA amplification products were quantified on a Qubit® 2.0 Fluorometer (Thermo Fisher Scientific, Walsham, United States) using the QubitTM dsDNA HS Assay kit (Thermo Fisher Scientific, Walsham, United States). Then use the Human Erythrocyte *RHD* Genotyping Kit (PCR-SSP) (Tianjin Super Biotechnology Developing Co., Ltd., Tianjin, China; Lot No. 190830001) to detect exon 1, 5, 6, 7, 9. The PCR amplification system consisted of dNTP-Buffer working solution 80 μL, Taq enzyme 0.8 μL, DNA 10 μL, and 10 μL of the above-mixed solution was added into each of the eight wells coated with primers. PCR amplification procedure: pre-denaturation at 96°C for 2 min; 96°C for 20 s, 68°C for 1 min, 8 cycles; 96°C 20 s, 65°C 50 s, 72°C 45 s, 11 cycles; 96°C 20 s, 62°C 50 s, 72°C 45 s, 30 cycles; Finally, it was extended at 72°C for 5 min. PCR amplification products were imaged on a gel imager after 2.5% agarose gel electrophoresis, and the results were interpreted RhD+ when any of the exons (1, 5, 6, 7, 9) were positive for *RHD* deletion women, and any of the exons (5, 6, 7, 9) were positive for RHD-CE(2-9)-D women.

## Results

### *RHD* Genotypes in D¯ Pregnancy Women

The PCR-SSP results indicated 63.2% (48/76) *RHD* deletion homozygous, 15.8% (12/76) *RHD* 1227A homozygous, 14.5% (11/76) RHD-CE(2-9)-D, 2.6% (2/76) *RHD* VI III, and 3.9% (3/76) genotypes could not be identified by PCR-SSP (Table 1).

**TABLE 1:** Genotyping results of *RHD* by PCR-SSP method in 76 pregnant women in Shaoxing.

Genotype	Number of samples	Proportion (%)
*RHD* deletion	48	63.2
RHD-CE(2-9)-D	11	14.5
*RHD* 1227A homozygous	12	15.8
*RHD* VI III	2	2.6
Undetectable	3	3.9
Total	76	100

### Three Cases of *RHD* Point Mutation

**FIGURE 1:**
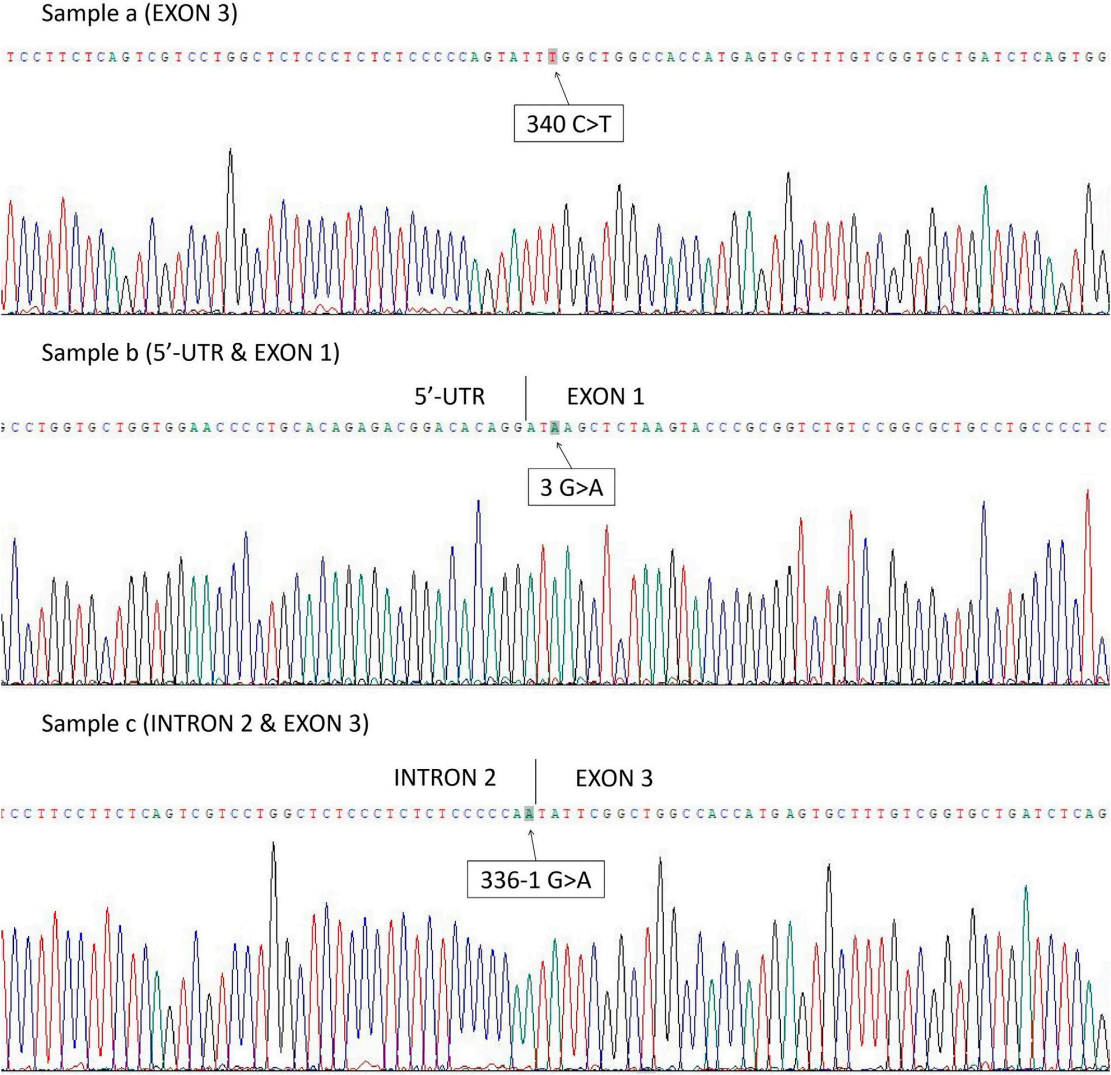
Three cases in which the genotype could not be confirmed by PCR-SSP method were found to have point mutations after *RHD* Sanger sequencing: Sample a. RHD*10.08 partial D type (exon 3 c.340C>T); Sample b. RHD*01EL.02 type (exon 1 c.3G>A); Sample c. RHD*01N.25 type (intron 2 c.336-1G>A).

The first case was RHD*10.08 partial D type (exon 3 c.340C>T) (Figure 1, Sample a). The second was RHD*01EL.02 type (exon 1 c.3G>A) (Figure 1, Sample b), and a mutation at the splicing site was found as RHD*01N.25 type (intron 2 c.336-1G>A) (Figure 1, Sample c) in the third case.

### Family Study of 336-1G>A Mutation

**FIGURE 2:**
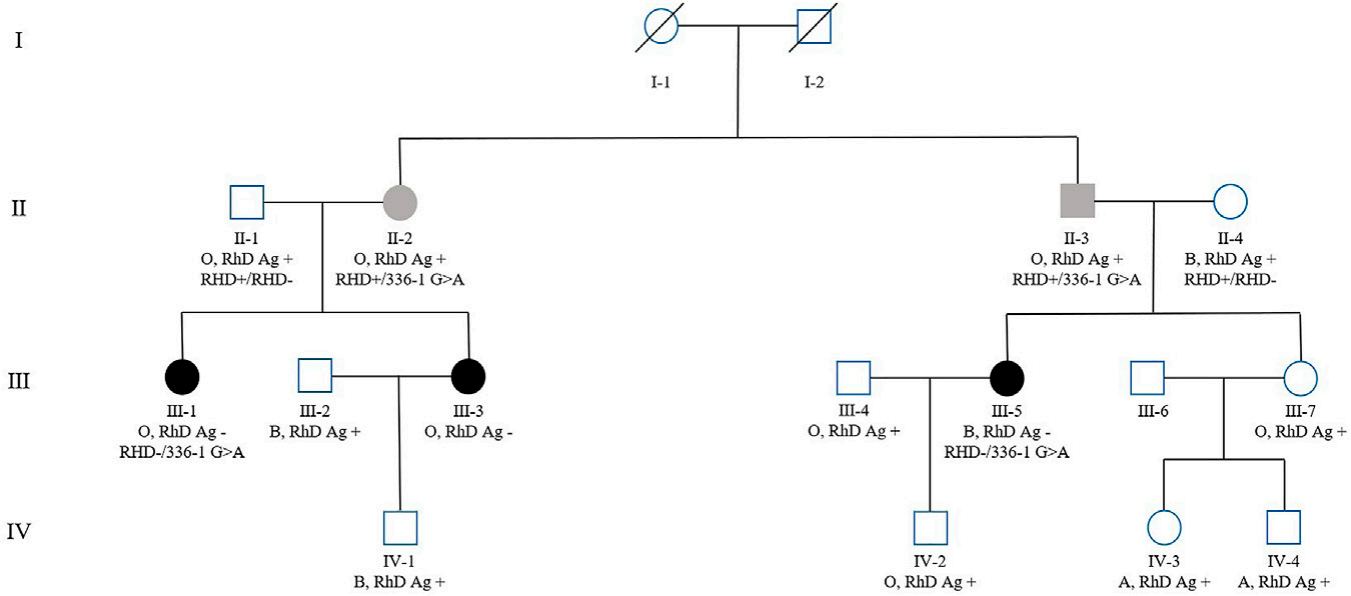
Family pedigree of the patient with RHD*01N.25 type (intron 2 c.336-1G>A), with ABO blood group, RhD antigen phenotype, and genotype (some family members were tested), labeled sequentially below each case in the pedigree.

Two other RhD-negative family members (III-1, III-3) were identified in the family of the patient with RHD*01N.25 (intron 2 c.336-1G>A) (Figure 2). PCR-SSP and *RHD* zygosity analysis confirmed the proband's mother (II-4) and uncle-in-law (II-1) had the hybrid Rhesus box detected, with *RHD*+/*RHD*- genotype (Supplementary Figures S1–S2). In comparison, proband's father (II-3), aunt (II-2) (Supplementary Figure S3) have the same genotype *RHD*+/336-1G>A, which speculated the zygosity of the proband and her cousin (III-1) as *RHD*-/336-1G>A. RhD serological results and *RHD* genotype of this family are shown in Table 2.

**TABLE 2:** *RhD* serological results and *RHD* genotype of the family of the case with RHD*01N.25 (intron 2 c.336-1G>A).

Subject	RhD saline test	Irregular antibodies	DAT	(Modified) IAT			Absorption and elution test (absorbed by IgG anti-D in a ratio of 1:1)	Serological RhD phenotype	*RHD* Genotype
				IgG anti-D	IgG + IgM anti-D^①^	IgG + IgM anti-D^②^			
Ⅱ-1	4+	0	0	-	-	-	-	D+	*RHD+/RHD-*
Ⅱ-2	4+	0	0	-	-	-	-	D+	*RHD+/*336-1G>A
Ⅱ-3	4+	0	0	-	-	-	-	D+	*RHD+/*336-1G>A
Ⅱ-4	4+	0	0	-	-	-	-	D+	*RHD+/RHD-*
Ⅲ-1	0	0	0	0	0	0	0	D−	*RHD-/*336-1G>A
Ⅲ-3	0	0	0	0	0	0	0	D−	
Ⅲ-5(proband)	0	0	0	0	0	0	0	D−	*RHD-/*336-1G>A
Ⅲ-7	+	0	-	-	-	-	-	D+	
*RHD* deletion homozygous	0	0	0	0	0	0	0	D−	
*RHD* 1227A homozygous	0	0	0	0	0	0	1+	Del	
*RHD+* homozygous	4+	0	0	4+	4+	4+	3+	D+	

Note: a The sample with RHD deletion homozygous and the sample with RHD+ homozygous were used as negative control and positive control for each test, respectively. The sample with RHD 1227A homozygous was used as weak positive control for absorption and elution test. b The results of III-7 were obtained from the records of delivery in our hospital, and the intensity of agglutination was not determined. c IgG + IgM anti-D ① and IgG + IgM anti-D ② are reagents of different batches from the same manufacturer. Abbreviations: DAT, direct antiglobulin test; IAT, indirect antiglobulin test.

### mRNA Sequence Analysis

**FIGURE 3:**
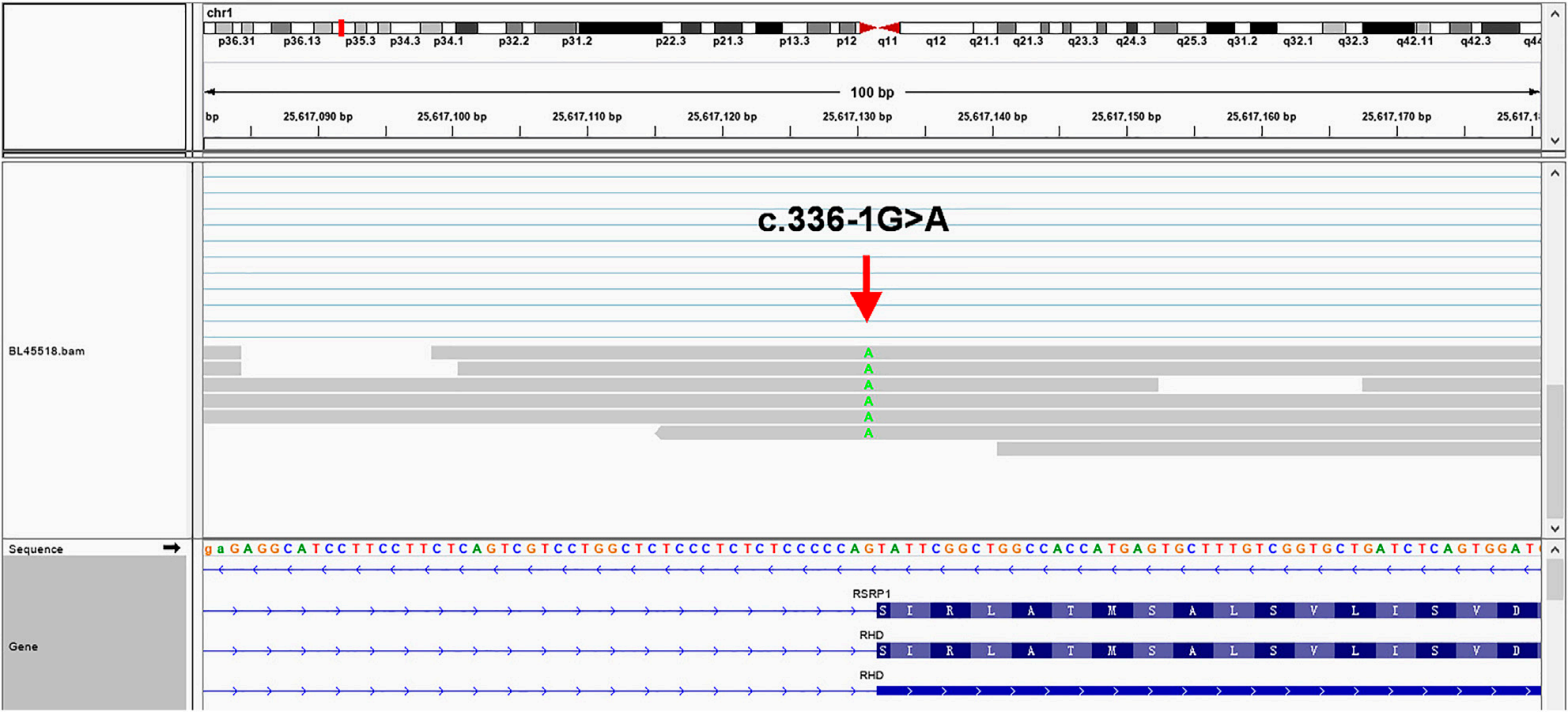
mRNA-seq results using the Integrative Genomics Viewer in a web browser (IGV-Web app version 1.6.3) (Robinson et al., 2011), showing multiple splicing products with different lengths (31 bp - 197 bp) of intron 2 sequences residue which are much longer than the 28 bp predicted by Fichou (Fichou et al., 2015), verifying that a cryptic site upstream of the constitutive acceptor site was activated in the presence of c.336-1G>A (green). To avoid the possibility of paralogs of transcript isotype, we BLAT the read's sequences in different regions and confirm that 197 bp intron 2 sequences residue near the mutation site is unique and has no isotypes.

The sequencing results (Figure 3) verified 336-1G>A mutation site and revealed the multiple splicing products of different lengths with the intron 2 residue. In addition, the number of reads in intron 2 was significantly higher than other introns (Supplementary Figures S4–S6), suggesting that intron 2 was improperly spliced after c.336-1G>A mutation.

### Non-invasive Fetal *RHD* Genotyping

Fetal RhD status in 28 cases of maternal homozygous *RHD* deletion and 3 cases of maternal RHD-CE(2-9)-D were predicted (Table 3). All predicted results were consistent with the newborn RhD status. Two cases showed RhD negative in the first samples but confirmed positive in the second samples.

**TABLE 3:** Fetus RhD status predicted results of 33 samples of NIPT plasma in 31 pregnant women.

		Maternal *RHD*-Deletion	Maternal RHD-CE(2-9)-D
Plasma samples Number		28	3
Predicted fetus RhD status	RhD-positive	28	3
	RhD-negative	0^a^	0
	Uncertain	0	0
Newborn RhD status	RhD-positive	28	3

a 2 cases show negative results in 12-16th gestation week, but results in the second samples after few weeks later show positive.

## Discussion

### *RHD* Genotypes of D¯ Pregnant Women in Shaoxing and the Molecular Diagnostic Strategy for Chinese D¯ Pregnant Women

**FIGURE 4:**
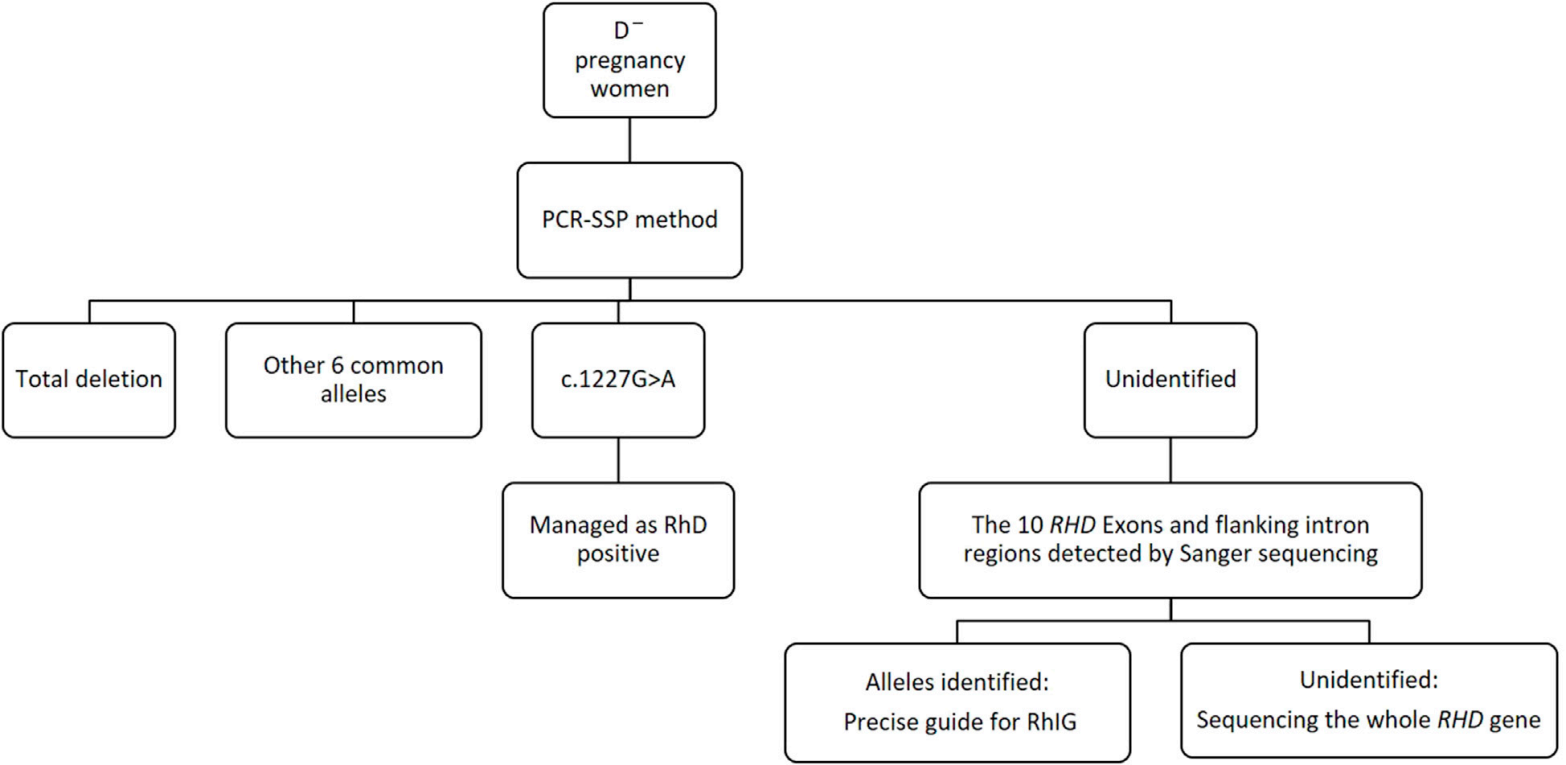
A simple decision tree for the molecular diagnosis of D^–^ pregnancy women in China.

RhD-negative frequencies show vast racial differences (Xu et al., 2003; Flegel et al., 2009; Polin et al., 2009; Cruz et al., 2012; Crottet et al., 2014; Perez-Alvarez et al., 2019) (Table 4). Thus, for diverse ethnic populations, it is necessary to adopt different *RHD* genotyping strategies to the spectrum of prevalent alleles. In our study, the main genotypes of *RHD* in D¯ pregnant women in Shaoxing were *RHD* deletion, *RHD* 1227A homozygous type, and RHD-CE(2-9)-D type. The profile of our survey was in concordance with that of another study in China (Zhang et al., 2019), indicating minor differences in the *RHD* genotypes of D¯ pregnant women across different regions in China. Using the PCR-SSP method, designed for eight common Chinese *RHD* genotypes: *RHD*-positive*, RHD* deletion, RHD-CE(2-9)-D, DVa (Hus), DVI III, weak D15, DEL *RHD* 1227A homozygous, and DEL *RHD* 1227A heterozygous, we found that about 96.1% of D¯ women can identify *RHD* genotype, and the rate rises to above 99% when adding the 10 *RHD* exons sequencing. Therefore, we established a simple molecular diagnostic strategy for Chinese D¯ pregnant women (Figure 4).

**TABLE 4:** Frequency of *RHD* alleles in serologic RhD-negative blood donors.

Country	Frequency (%)	Reference
German	0.21	Flegel et al. (2009)
Austrian	0.4	Polin et al. (2009)
Swiss	0.47	Crottet et al. (2014)
United States	0.94	Perez-Alvarez et al. (2019)
Brazilian	9.2	Cruz et al. (2012)
China	19.9	Xu et al. (2003)

### The Precise Guide of RhIG use for D¯ Pregnant Women

The occurrence of alloimmunity in D¯ pregnant women is related to several factors, such as maternal *RHD* genotype and RhD antigen epitope (Flegel, 2011); quantity of D-positive fetal red blood cells entering the body (Woodrow, 1971); and other factors such as ABO blood type, maternal HLA class, or fetal sex (Hadley and Soothill, 2002). However, the main factor is maternal *RHD* genotype and RhD antigen epitope. Therefore, our study guides RhIG use according to the different *RHD* genotypes in D¯ pregnant women (Table 5).

**TABLE 5:** *RHD* alleles and guidance for managing transfusion or RhIG administration.

Detected alleles in our study		ISBT allele designation	Candidate for RhIG	Suggested RhD phenotype as a donor	Suggested RhD phenotype as a recipient
RHD deletion		*RHD* *01N.01	Yes	Negative	Negative
RHD-CE(2-9)-D		RHD*01N.03	Yes	Negative	Negative
*RHD*(c.1227G>A)		RHD*01EL.01	No	Positive	Positive
*RHD* VI III		RHD*06.03.01	Yes	Positive	Negative
*RHD*(c.340C>T)		RHD*10.08 or RHD*01W.17	Yes	Positive	Negative
*RHD*(c.3G>A)		RHD*01EL.02	Yes	Positive	Negative
*RHD*(c.336-1G>A)		RHD*01N.25	Yes	Positive	Negative
**Alleles managed as D positive type in guidance (Flegel et al., 2020)**					
c.809T>G	RHD*01W.1		No	Positive	Positive
c.52C>G	RHD*01W.1.1		No	Positive	Positive
c.712G>A	RHD*01W.1.1		No	Positive	Positive
c.1154G>C	RHD*01W.2		No	Positive	Positive
c.301T>A	RHD*01W.2.1		No	Positive	Positive
c.916G>A	RHD*01W.2.2		No	Positive	Positive
c.932A>G	RHD*01W.2.2		No	Positive	Positive
c.8C>G	RHD*01W.3		No	Positive	Positive
c.178A>C	RHD*01W.3.1		No	Positive	Positive
c.602C>G	RHD*09.03.01		Yes	Positive	Negative
c.667T>G	RHD*09.03.01		Yes	Positive	Negative
c.819G>A	RHD*09.03.01		Yes	Positive	Negative
c.48G>C	RHD*09.04		No	Positive	Positive
c.819G>A	RHD*09.04		No	Positive	Positive

The *RHD* (c.1227G>A) allele, named the DEL type or Asian DEL type, is the most prevalent DEL allele in Asians. Asian DEL is always mistyped as D-negative by routine serological assays (Kim et al., 2020). Therefore, *RHD* genotyping is the gold standard for detecting DEL (Nuchnoi et al., 2014). The *RHD* (c.1227G>A) should be managed as D-positive for RhIG administration or selection of blood components for transfusion (Chun et al., 2020). In 2015, a study revealed that erythrocytes carrying the 1227A mutation might express very low “normal” D antigen levels (Fichou et al., 2015). This theory explains why “Asian DEL types” do not cause hemolytic disease in newborns and fetuses. Thus, at least 15% of D¯ pregnant women in China, typed as the *RHD* (c.1227G>A) allele, will benefit from *RHD* genotyping and be free from unnecessary RhIG use. At the same time, to avoid transfusion of DEL RBC units to D¯ recipients, RBC units carrying c.1227G>A mutation should be transferred into the D+ pool (Flegel et al., 2009).

RHD-CE(2-9)-D is a hybrid allele that replaces exons 2 to 9 by *RHCE*. Lacking D antigen expression, RBCs of RHD-CE(2-9)-D will not risk D¯ recipients (Flegel et al., 2009). At the same time, women with RHD-CE(2-9)-D should be managed as D¯ and be given RhIG for D immunoprophylaxis in pregnancy (Table 5).

DVI is the most common partial D that produces anti-D, and newborns born to DVI mothers with anti-D may develop hemolytic disease. DVI III is named a D-Ce(3-6)-D hybrid and DVI III erythrocytes carry relatively high RhD antigen densities. Therefore, DVI III recipients should be transfused with RhD negative blood, while DVI III donor should be managed as D^+^ (Wagner et al., 1998).

Our study characterized two point mutations in exons: c.340C>T and c.3G>A. According to the guideline (Flegel et al., 2020), both should be managed as D¯ and given RhIG for D immunoprophylaxis. More interesting, a recent study showed that weak-D and Asia-type DEL alleles’ coexistence would completely express the D-antigen (Chun et al., 2020), which means free from RhIG administration.

For our study’s 336-1G>A mutation, the mRNA products of the proband showed long intronic segment retention, which will induce DEL or D-negative phenotypes (Fichou et al., 2015). The serological study for family members reveals D¯ phenotypes in *RHD*-/336-1G>A heterozygote and D positive in *RHD*+/336-1G>A heterozygote (Table 2). However, lacking the detailed evidence for no D-antigen in red blood cells, we still suggested these individuals with *RHD*-/336-1G>A heterozygote should be managed as RhD positive donors in transfusion in an abundance of caution.

Thus, in our study, *RHD* genotyping for D¯ pregnant women eliminates at least 15% unnecessary ante- and postpartum injections of RhIG and gave a detailed guide of RhIG use for these D¯ pregnant women.

### c.336-1G>A Mutation Analysis and Family Study

More than 300 variant alleles of *RHD* have been reported (Kawano et al., 1998; Ye et al., 2007; Fichou et al., 2015; Chen et al., 2016; Ogasawara et al., 2016; El Wafi et al., 2017; Chun et al., 2018; Raud et al., 2019), including single-nucleotide polymorphisms (SNPs), small or large fragment deletions, gene rearrangements, and complete *RHD* deletions. The intron mutation disrupts a constitutive splice site, resulting in improper retention of an intron or activation of a cryptic splice site in the vicinity of the mutant. Thus, this mutation will induce DEL or D-negative phenotypes by minute to no expression of the D antigen at the surface of RBCs. As many intronic mutated alleles of *RHD* were identified in Sequencing, several bioinformatics tools were used to predict the defect resulting from a genetic variation in silico. However, very few functional data are available to confirm these predictions. Furthermore, lacking fresh blood samples for RNA extraction, many functional studies mainly relied on recombinant plasmids. Hence, clinical transcriptome data and protein analysis are urgently needed to investigate genotype-phenotype mechanisms (Liu et al., 2010; Fichou et al., 2015; El Wafi et al., 2017).

We found a family with 336-1G>A intron mutation in our study. The mutation site of 336-1G>A is an intron 2 acceptor. The 336-1G>A mutation was first reported in Korea in 2005 (Kim et al., 2005) and first identified in Chinese blood donors in 2009 (Ye et al., 2009), named as the RHD*01N.25 type (IVS2-1G>A) by ISBT. In 2015, The first functional analysis by plasmid recombination experiments revealed that the cryptic splicing site of c.336-1G>A was activated, and mRNA product was either increased by 28 base pairs (bp) (from intron 2) or decreased by 21 bp (exon 3) (Fichou et al., 2015).

Our serological D-negative proband was an *RHD-*deletion/336-1G>A heterozygote in our study. SpliceAI showed a 94% decrease in the probability of acting as a splice acceptor in the 336-1G>A site. Our mRNA products of the 336-1G>A mutation under physiological conditions confirmed the prediction. A cryptic site upstream of the constitutive acceptor site was activated in the presence of c.336-1G>A, which resulted in insertion of an intronic sequence within the mature transcript. The retentional intronic segments (31 bp–197 bp) are much longer in our study than the 28 bp predicted by Fichou (Fichou et al., 2015), which suggests that the mRNA product under physiological conditions is different from the plasmid recombination experiments. This mature *RHD* transcript with intronic segments might be translated into a protein with an additional 10–66 amino-acid sequence, which may alter the structure of the second extracellular loop of RhD. We didn't identify the active cryptic splice site in intron 2. However, we supposed the intronic retention inducing in-frame shift because the intron 3 seems correctly splicing in our study. All reported *RHD*-/336-1G>A cases are completely serological D-negative, which means no antigens can be detected (Kim et al., 2005; Ye et al., 2009; Fichou et al., 2015). Our results first revealed that the 336-1G>A intron mutation induces the retention of intron 2, which will generate improper peptides.

### Fetal *RHD* Genotyping in Maternal Plasma

Prenatal detection of the fetal RhD status is helpful to assess the risk of hemolytic disease in fetus. Non-invasive prenatal testing for predicting fetal RhD status is available in many countries (Finning et al., 2008; Akolekar et al., 2011; Wikman et al., 2012; Chitty et al., 2014; Clausen et al., 2014). The standard quantitative PCR method is based on the assumption of homozygous *RHD* deletion in D¯ pregnant women. *RHD* variants in D¯ women will yield high false-positive results (Yang et al., 2019). Therefore, Non-invasive fetal *RHD* genotyping is only feasible for D¯ women with total or partial deletion of the *RHD* (Clausen et al., 2019; Yang et al., 2019).

Unlike the high-throughput quantitative PCR method widely used in European countries, we use the PCR byproduct from routine NIPT for aneuploidy as DNA template, then amplified by PCR-SSP method. Because most Chinese pregnant women choose NIPT for aneuploidy after 12 gestational weeks, our NIPT + PCR-SSP protocol for D¯ pregnant women is cost-effective and suitable for the small number of D¯ samples in China. In addition, the results of routine NIPT for aneuploidy can provide good quality control data, such as fetal fraction concentration.

Many research chooses the exons 5, 7, 10 as the targeted exons to predict fetal RhD status (Finning et al., 2008; Akolekar et al., 2011; Chitty et al., 2014; Clausen et al., 2014), while Wikman (Wikman et al., 2012) choose exon 4 as target exon. However, our study found that any one of exon 1, 5, 6, 7, and 9 positive can predict fetal D+ status. Furthermore, all our predicted results were consistent with the newborn RhD status.

Our study also emphasized the importance of resampling for the first negative results. Two cases showed negative results in the first sample but were later confirmed as positive in the second sample, which was likely due to the lower fetal fraction content or PCR bias (Sabina and Leamon, 2015). Thus, we argued the second sampling after a few weeks to confirm the first negative results (Table 3).

No fetuses were predicted as RhD negative in our study. Asian D-negative pregnant women have a 96% possibility of having a D-positive fetus (Chun et al., 2020). Predicting fetal RhD status is not cost-effective in China, whatever methods are used.

### Limitation of Our Study

To our knowledge, our study used the mRNA-seq to analyze the splicing products of the 336-1G>A under physiological conditions at the first time. However, there has a significant limitation by using an inappropriate sample type, whole peripheral blood, instead of enriched reticulocytes. Hence the reads numbers are relatively small in mRNA-seq. The active cryptic site upstream of the constitutive acceptor site is not identified in our study.

## Conclusion

In conclusion, we established a pathway to precisely manage Chinese D¯ pregnant women, including using the PCR-SSP method and Sanger sequencing to provide precise RhIG administration and promote transfusion safety. The robust protocol for determining fetal RhD status from maternal plasma is suitable for Chinese D¯ women. In addition, the detailed study on rare alleles will help us investigate the molecular mechanisms between genotype and phenotype of *RHD* variations.

## Data Availability

1) The data presented in the study are deposited in the China National GeneBank DataBase (CNGBdb) repository, accession number CNP0002279, could be founded with https://db.cngb.org/cnsa/project/CNP0002279/reviewlink/. 2) The genetic data could not be publicly available because of (Regulation of the People’s Republic of China on the Administration of Human Genetic Resources), any inquries could contact the corresponding author for details.
