# Supplementary material for: RHD Genotypes in a Chinese Cohort of Pregnant Women
Source: Front Genet. 2021 Dec 14;12:752485. doi: 10.3389/fgene.2021.752485 (PMC8712876; doi:10.3389/fgene.2021.752485)
Supplement: Supplementary file 1 [file DataSheet1.docx]

Supplementary Material

II-1


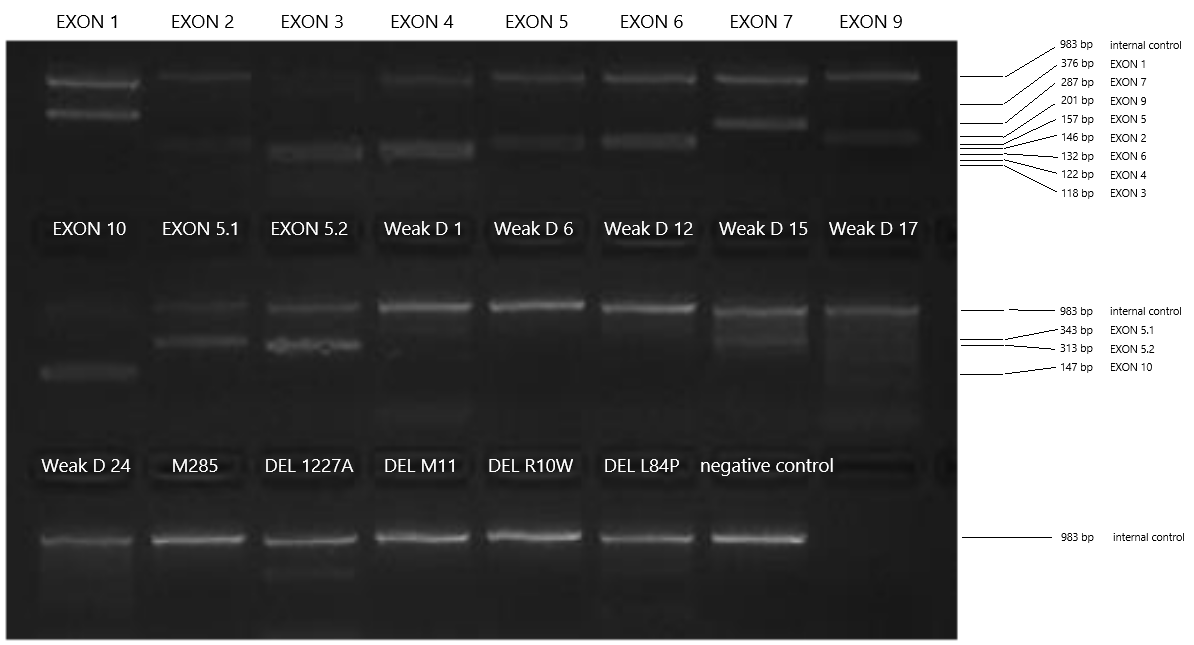


II-4


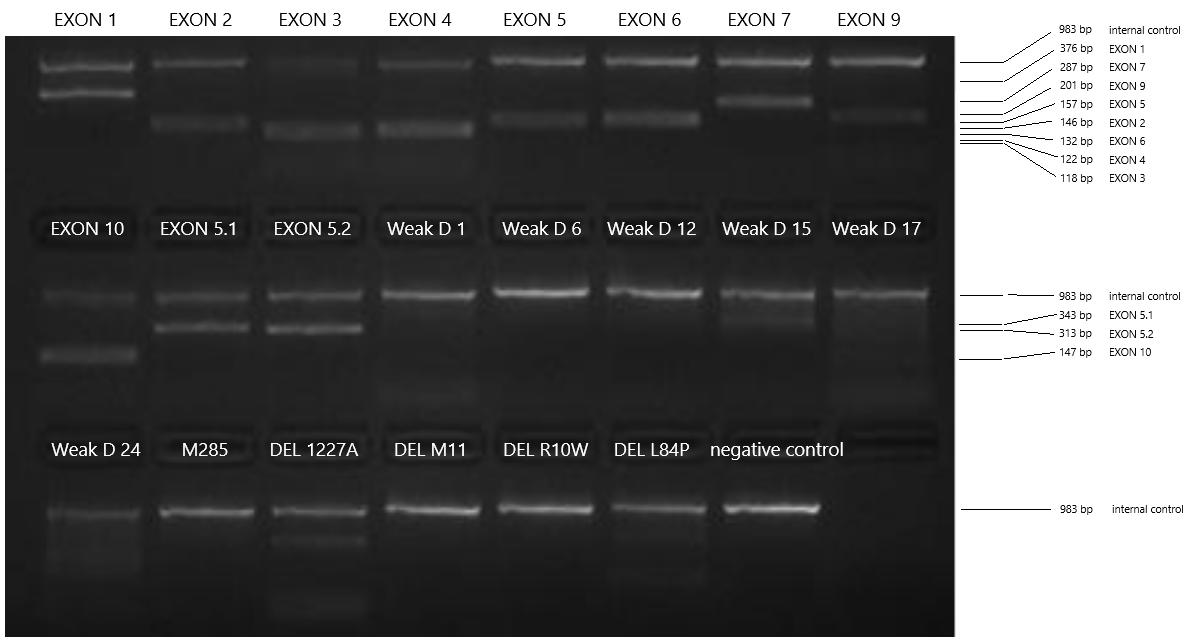


Figure S1 The PCR-SSP results of family members (II-1 and II-4) of the proband with *RHD*01N.25* (intron 2 c.336-1G>A), verifying that both have normal *RHD* gene on at least one chromosome. In both samples, *RHD* specific PCR products are obtained for the *RHD* exon 1 (376 bp), exon 2 (146 bp), exon 3 (118 bp), exon 4 (122 bp), exon 5 (157 bp), exon 6 (132 bp), exon 7 (287 bp), exon 9 (201 bp), exon 10 (147 bp), exon 5.1 (343 bp) and exon 5.2 (313 bp). The length of internal control product is 983 bp.


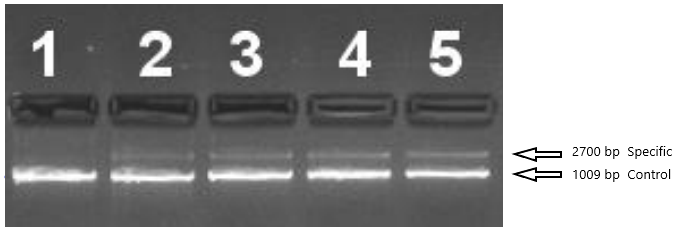


Figure S2 The zygosity results of family members (II-1 and II-4) of the proband with *RHD*01N.25* (intron 2 c.336-1G>A), both of whom have hybrid Rhesus box detected, verifying *RHD+/RHD-* genotype. The band close to the well represents a specific positive result, i.e., hybrid Rhesus box of *RHD-* (2700 bp) is detected, while the band far away from the well is an internal control (1009 bp). 1: negative control (normal *RHD* gene on both chromosomes); 2-3: positive control (*RHD* deletion homozygous); 4: zygosity result of II-1; 5: zygosity result of II-4.

II-2


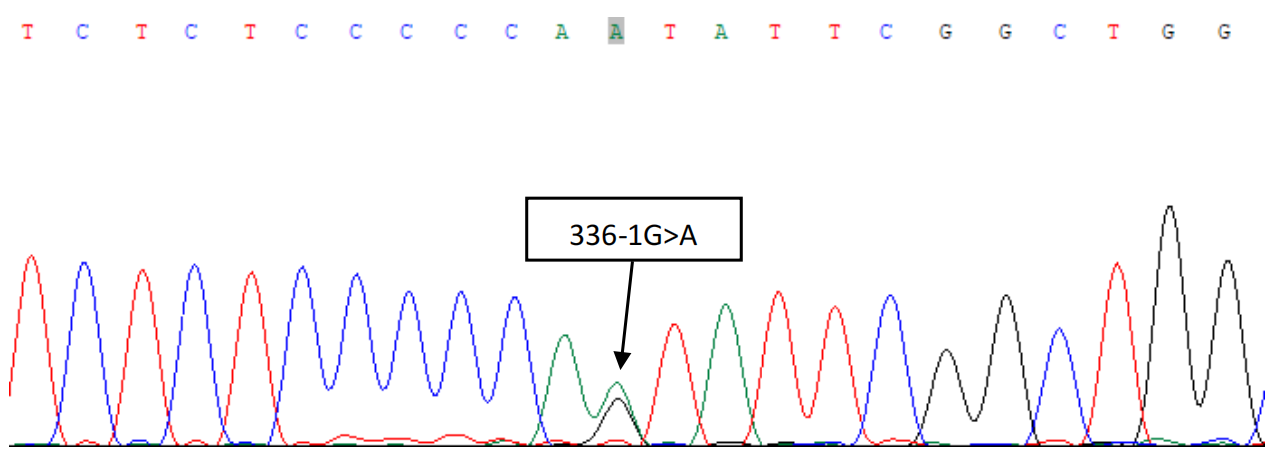


II-3


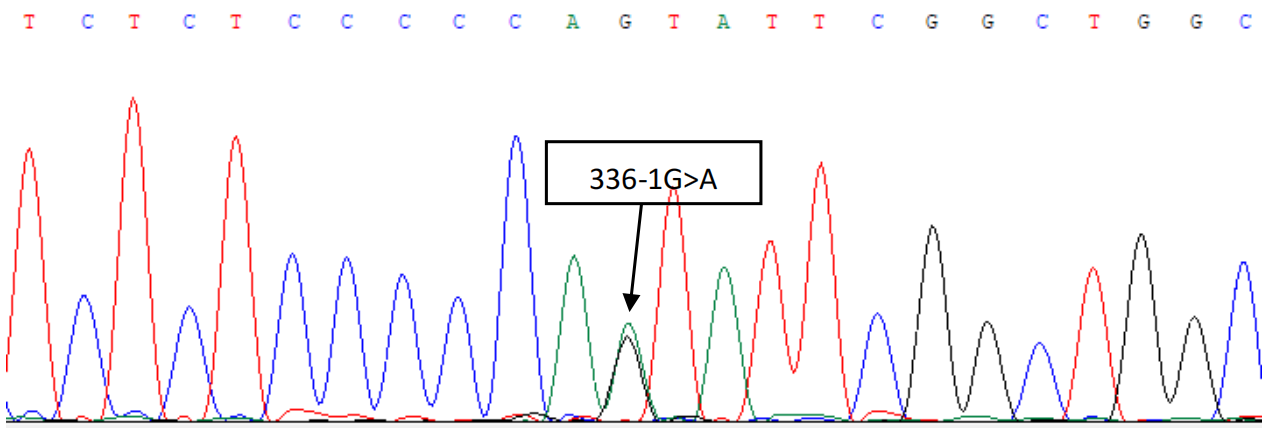


III-1


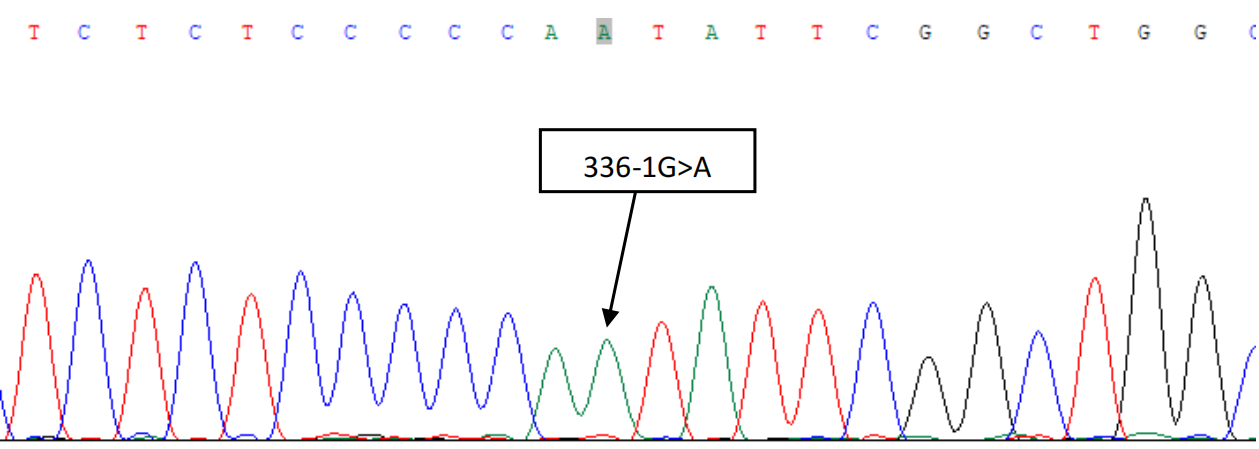


Figure S3 *RHD* gene Sanger sequencing results of family members (II-2, II-3, III-1) of the proband with *RHD*01N.25* (intron 2 c.336-1G>A), whose genotype is *RHD+*/336-1G>A, *RHD+*/336-1G>A and *RHD-*deletion/336-1G>A, respectively.


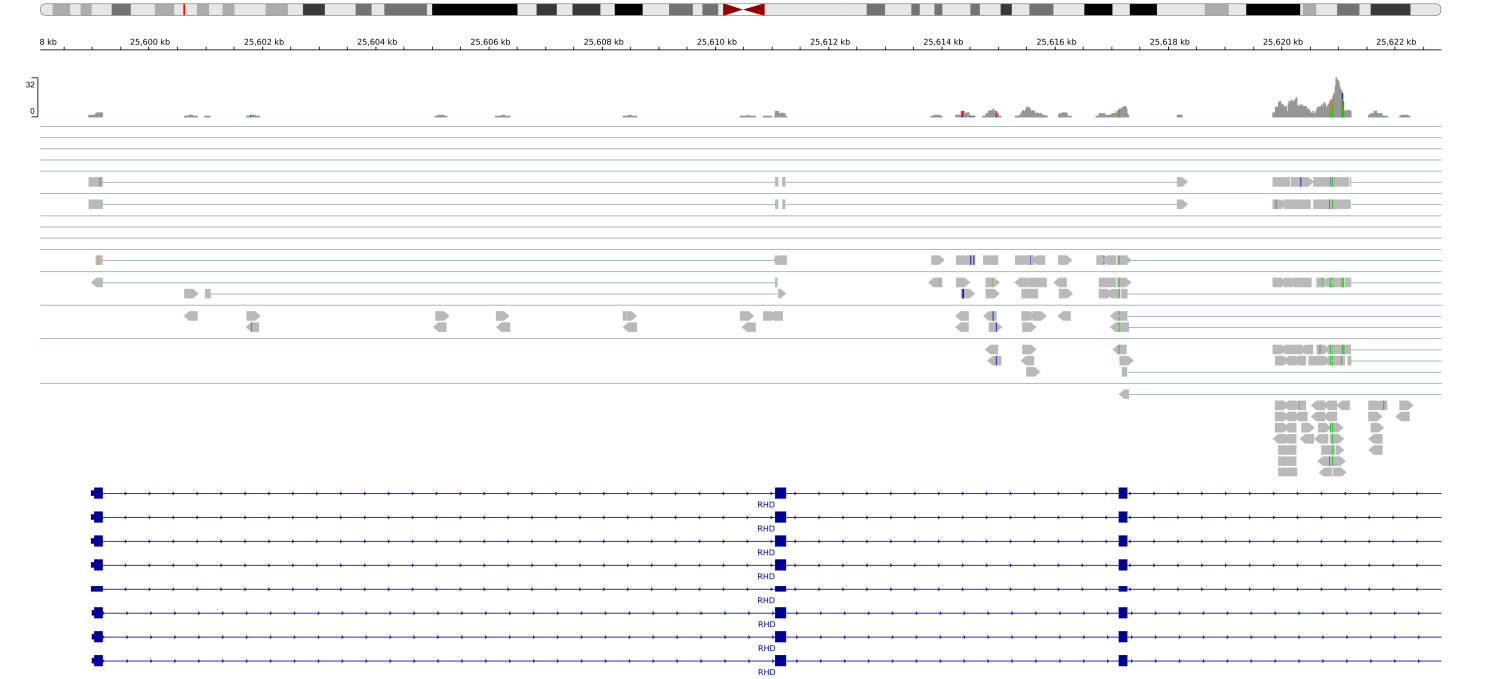


Figure S4 Reads distribution of exons 1-3 of *RHD* gene in mRNA-seq results using the IGV-Web app. To avoid the possibility of paralogs of transcript isotype, we BLAT the read's sequences in different regions. Thus, 197 bp intron 2 sequences residue near the c.336-1G>A mutation is unique and has no isotypes. In comparison, the reads in intron 3 and intron 2 (far from the mutation site) have many isotypes. Thus, we only confirmed the intron 2 retention in our study.


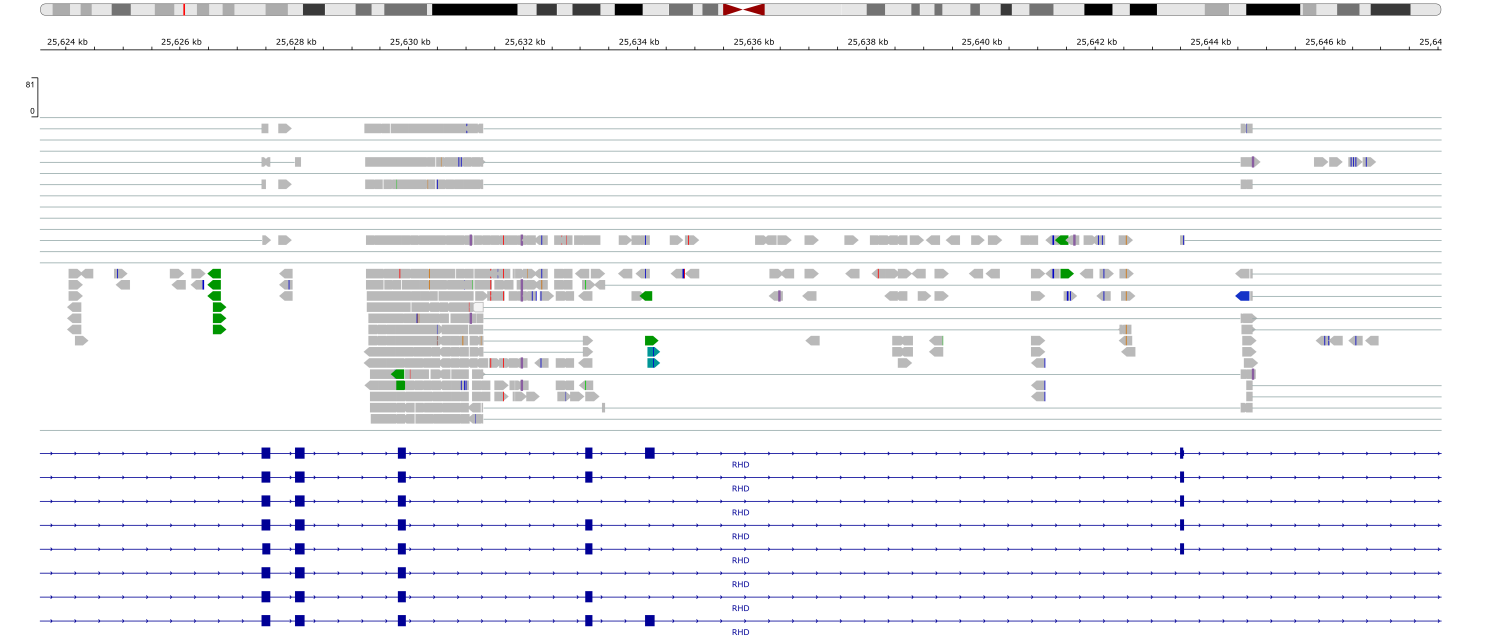


Figure S5 Reads distribution of exons 4-8 of *RHD* gene in mRNA sequencing results using the IGV-Web app.


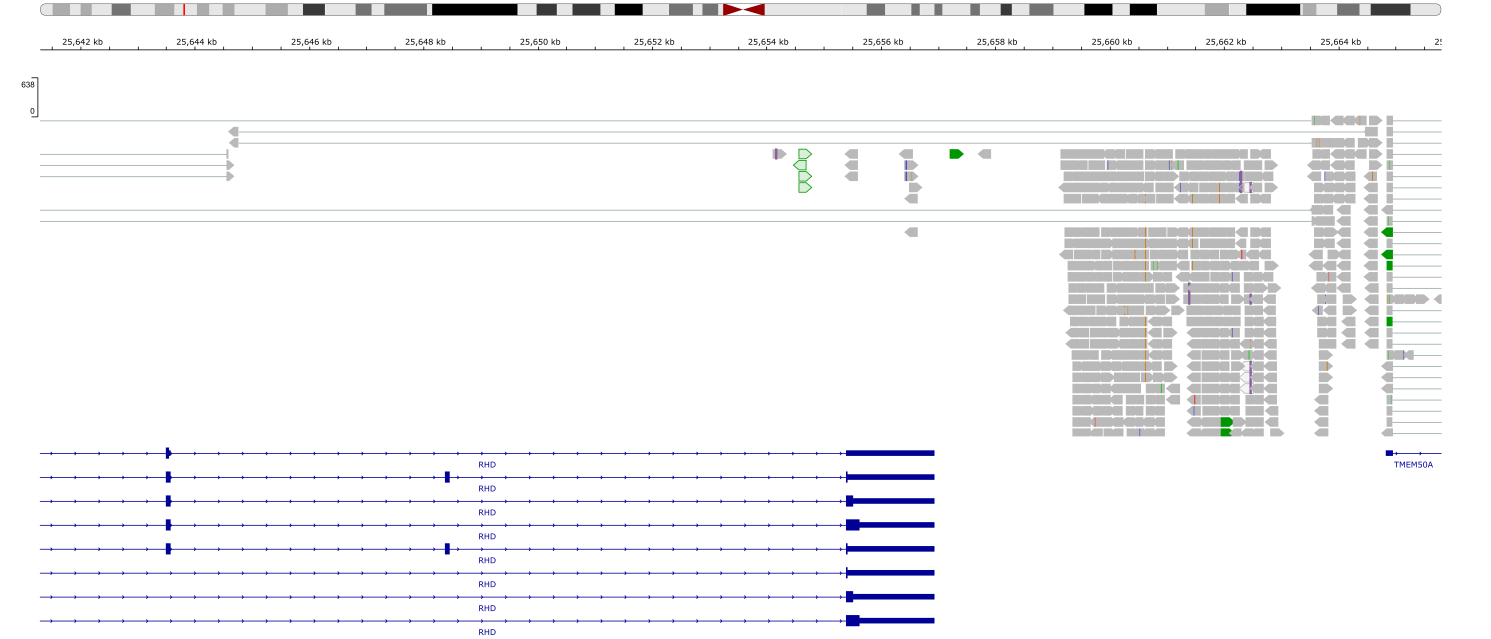


Figure S6 Reads distribution of exons 8-10 of *RHD* gene in mRNA sequencing results using the IGV-Web app.
